# Zika virus M protein latches and locks the E protein from transitioning to an immature state after prM cleavage

**DOI:** 10.1038/s44298-023-00004-2

**Published:** 2023-11-06

**Authors:** Sydney A. Majowicz, Anoop Narayanan, Ibrahim M. Moustafa, Carol M. Bator, Susan L. Hafenstein, Joyce Jose

**Affiliations:** 1 The Huck Institutes of the Life Sciences, The Pennsylvania State University, University Park, PA 16802 USA; 2 Department of Biochemistry and Molecular Biology, The Pennsylvania State University, University Park, PA 16802 USA; 3 Department of Medicine, The Pennsylvania State University College of Medicine, Hershey, PA 17033 USA

**Keywords:** Virology, Virus structures

## Abstract

During flavivirus maturation, the structural proteins prM (pre-membrane) and E (envelope) undergo extensive low pH-mediated conformational changes, transitioning from spiky trimeric to smooth dimeric prM/E heterodimers which allow for furin cleavage of prM into pr and M and forms the irreversible mature conformation of smooth M/E heterodimers. The mechanisms of irreversible conformational changes to E protein following the pr cleavage are not understood. Utilizing cryo-EM structures of immature virus and structure-based mutagenesis of Zika virus, we identified two critical “latching and locking” interactions mediated by M protein residues Arg38 and Trp19, respectively, that stabilize the E protein structure in the smooth mature stage. M protein thus latches and locks the E protein in an irreversible mature structure, preventing premature fusion in the secretory pathway. Our studies provide mechanistic insights into the reversible structural transition of immature trimeric spikes and the irreversible transition of smooth dimeric M/E heterodimers critical for virus infectivity.

## Introduction

Zika virus (ZIKV) is a mosquito-borne flavivirus in the *Flaviviridae* family associated with Guillain-Barré syndrome in adults and congenital Zika syndrome in newborns^1^. Among flaviviruses, ZIKV has been reported to have a unique ability of human-to-human sexual transmission and maternal-fetal transplacental transmission during pregnancy, which has been associated with fetal demise and microcephaly^2–5^. ZIKV was maintained at low levels in the Eastern hemisphere until the first human epidemic occurred on Yap Island in 2007^6^. Later, ZIKV infection was detected in Brazil in 2015, and the virus spread in the Americas, where cases peaked in 2016^7–9^. Since the detection of ZIKV in Brazil, 45 countries in the Americas have reported local ZIKV transmission with 200,000 infections, of which 3000 manifested congenital Zika syndromes such as microcephaly and other abnormalities in the developing fetus and newborns^8,10,11^. Although human infections have since declined, ZIKV remains a significant public health problem due to continued spread, the ability of human-to-human transmission, and the lack of approved antiviral therapies and vaccines^12,13^.

Flaviviruses have a single-stranded, positive sense, ~11 kb RNA genome encoding three structural (C, prM, E) and seven non-structural (NS1, NS2A, NS2B, NS3, NS4A, NS4B, NS5) proteins^14^. Electron tomography studies have indicated that viral RNA replication occurs in association with vesicle packets on the endoplasmic reticulum (ER) membrane that are connected to the cytoplasm via a pore through which the nascent positive-strand RNA exits, binds C (capsid) protein in the cytoplasm, and initiates nucleocapsid assembly^14–16^. The genomic RNA bound to several copies of C protein form a nucleocapsid core that buds into the ER lumen, acquiring the viral envelope from the ER membrane containing 60 icosahedrally arranged trimeric spikes of prM and E heterodimers forming a 60 nm diameter immature virus^17–20^. Immature virus transits through the acidic compartments of the secretory pathway, which induces significant conformational changes to the prM/E, allowing the cleavage of prM into pr and M by a host furin-like serine protease in the late-Golgi to produce the 50 nm mature virus that exits the cell through the secretory pathway^14,21,22^. The mature virus contains 180 copies of the M and E proteins icosahedrally arranged as 90 dimers of M and E heterodimers in a herringbone pattern^23,24^. Although an icosahedrally ordered nucleocapsid core is not resolved in the mature flavivirus structures, cryo-EM structures of immature ZIKV, dengue virus (DENV), and Kunjin virus (KUNV) have indicated the presence of a partially ordered C protein shell^19,20,25^. Furthermore, densities corresponding to the C protein connecting the membrane proteins identified in the immature virus cryo-EM structures provide evidence for interactions involved in virus budding and maturation-related rearrangements of the nucleocapsid^20,25,26^.

Receptor binding, entry, and membrane fusion are mediated by the E protein, which has three ectodomains (E-DI, E-DII, and E-DIII) followed by three perimembrane helices (E-H1, E-H2, and E-H3) and two transmembrane helices (E-T1 and E-T2)^27–31^. DII contains a fusion loop at its tip that inserts into the host-cell membrane for drawing host-cell and viral membranes together during entry^27,32^. DI is connected to DIII by a single peptide strand, whereas DI and DII are joined by four peptide strands that function as a molecular hinge, providing flexibility for low-pH mediated conformational changes of E^27,33^. The fusion peptide is buried in a cavity formed by DI and DIII from the adjacent E molecule in the mature virus^28,34^. M protein has an extended N-terminal loop, an amphipathic perimembrane helix (M-H), and two transmembrane helices (M-T1 and M-T2)^35,36^. Unlike the immature virus, the E stem region of the mature virus harbors two lipid-like ligand binding sites: the S1 site between E-H1 and E-H2 and S2 between E-H1, E-H2, and E-H3^35^ and an S3 site between the transmembrane helices of E (E-T1) and M (M-H2)^23,34^.

In the immature virus, the prM protein acts as a molecular chaperone, protecting the fusion loop of E by pr domain binding and preventing premature fusion of the E protein with host cell membranes triggered by low pH^18,21,22,36,37^. The prM polypeptide chain extends linearly along the surface of the E protein inside the spike at neutral pH, making the furin cleavage site inaccessible to host furin^18,19,21,22,35^. Subsequently, the immature spikes undergo a low pH-mediated rearrangement, transitioning from 60 trimeric prM/E heterodimers with a sequestered furin site to 90 prM/E heterodimers in a folded back “smooth” immature structure with an exposed cleavage site, enabling furin cleavage^18,21,22,32^. After furin cleavage, the cleaved pr remains bound to the E protein of the mature virus at low pH, effectively preventing premature membrane fusion by shielding the fusion loop, and the pr is shed at neutral pH when the mature virus is released via exocytosis^21,22,32^. Without the furin cleavage of prM, the smooth immature virus reverts to a spiky conformation at neutral pH, and non-infectious immature particles are released^22^. The low pH-induced conformational changes of the immature virus are reversible as long as prM remains uncleaved, suggesting that the cleaved M protein is essential for the irreversibility of the maturation process^18,21,22^. Although the structural information of amino acid interactions that mediate the maturation process is beginning to unfold, the molecular interactions driving the irreversible structural transition of the mature virus have not been established.

Several elegant structural studies and structure-based functional analyses have provided models for the immature to mature structural transition of flaviviruses, however, the structural intermediates and the effect of local environments such as temperature and pH in the mammalian host and arthropod vectors that define these global transitions remain incompletely understood^31,38–40^. Initially, a “draw string” model was proposed based on the cryo-EM structure of DENV to explain the spiky to smooth transition, which suggested the protonation of histidine residues of prM and E at low pH leads to the tightening of the pr linker causing a downward pull on the attached pr and the E protein binding to the pr domain^36^. Recently, based on the 3.8 Å resolution cryo-EM structure of immature Spondweni virus (SPOV), an alternate “pillar-collapse” model for flavivirus maturation has been proposed, which suggests the protonation of the histidine residue on prM linker releases the E protein from a strained, immature spiky conformation, leading to the “collapse” of E into a smooth, relaxed conformation^18^. Another immature virus cryo-EM structure of an insect-specific Binjari virus (BinJV) at 4.4 Å has revealed the complete structure of the prM linker^17,41^. As previously determined for the DENV prM/E structure, the organization of the BinJV prM/E heterodimer confirmed that the furin cleavage site is buried within the spike, which supports a maturation model that suggests that following the acidification of the prM linker histidine, the centrally placed prM guides the collapse of the spike along a pre-determined path^17,21^. Although the immature structures of BinJV and SPOV provide improved resolution of the prM/E heterodimer, the organization of the nucleocapsid and the densities connecting the trimeric spike with the nucleocapsid remain unresolved, presumably because these structures were generated by selecting immature virus particles from a mature preparation containing a mixture of mature and immature particles. Moreover, it is probable that the immature structures of BinJV and SPOV already represent viruses that have undergone a low pH-mediated structural rearrangement of the nucleocapsid; hence, they are not identical to the previously resolved neutral pH 9 Å resolution immature structures of DENV and ZIKV which have discernable nucleocapsid densities^19,20^.

Here, we generated an 8.3 Å resolution cryo-EM structure of immature ZIKV at neutral pH that facilitated structure-function studies and provide a new “latch and lock” mechanism that outlines two critical steps that control the low pH mediated reversible and maturation mediated irreversible transition into a metastable virion poised to undergo low pH mediated fusion upon entry into new cells. Using extensive structure-based mutagenesis, we characterized the functional role of amino acids at the prM/E and M/E interface and identified two critical sets of interactions. First, the electrostatic “latching” interaction that governs the reversible spiky to smooth transition involving M R38, E E216, and E D220 allows the formation of smooth immature structure and subsequent exposure of furin cleavage site at low pH. The second set of hydrophobic “locking” interactions involving M W19, E W211, and E L269 at the E-E dimer interface can only occur after the furin cleavage, release, and rearrangement of N-terminal loop of the M protein. Therefore, the deprotonation of histidine residues that generally triggers the hinge movement and transition of E protein causing premature fusion is averted concurrently by the locking interactions that prevent E protein from rising and masking the E protein fusion loop by the cleaved pr, which, when released allows the E protein fusion and post-fusion trimer formation during subsequent infection. Therefore, our studies provide mechanistic insights into molecular interactions that govern the maturation-related structural rearrangements required for the formation and stability of ZIKV mature virion.

## Materials and methods

### Cells and viruses

Vero E6 (ATCC, CRL-1586), HEK 293 T (ATCC, CRL-3216), and Huh 7.5 (a kind gift from Charles M. Rice) cells were grown and maintained at 37 °C in the presence of 5% CO_2_ in Dulbecco’s Modified Eagle’s Medium (DMEM, Gibco, 12800-082) supplemented with 10% fetal bovine serum (FBS, Seradigm, 1500-500), non-essential amino acids (NEAA, HyClone, SH30238.01), and penicillin streptomycin solution (PS, Corning, 30-002-CI).

### Purification of immature Zika virus

Immature ZIKV particles were purified from infected cells treated with NH_4_Cl, as described previously^42^. Briefly, Vero E6 cells were grown in DMEM supplemented with 2% FBS, NEAA, and PS and infected with ZIKV at an MOI of 0.1. After 24 h of incubation, the media was replaced with DMEM supplemented with 2% FBS, NEAA, PS, and 50 mM NH_4_Cl. Cell culture supernatant was collected after 48 h of incubation and clarified by centrifugation at 5000 × *g* for 15 min. PEG8000 at a final concentration of 8% was added to the supernatant and incubated at 4 °C with slow mixing for 4 h. The precipitate was spun down at 9500 × *g* for 50 min at 4 °C, re-suspended in TNE buffer (25 mM Tris pH 7.4, 100 mM NaCl, 0.5 mM EDTA), and centrifuged at 500 × *g* for 15 min to remove undissolved precipitates. The clarified solution was subjected to density gradient ultracentrifugation on a 10–35% tartrate gradient. The virus band was collected, buffer exchanged, and concentrated for vitrification using a 100 kDa cutoff concentrator (Merck Millipore Ltd., UFC810024).

### Cryo-EM structure determination and data collection

A purified virus sample was first assessed by negative staining TEM to check sample quality and concentration before preparing grids for data collection. Briefly, a 3.5 µL aliquot was applied to freshly glow-discharged Cu-grid coated with a thin film of continuous carbon, washed, stained with 0.75% uranyl formate for 15 s, blotted, air-dried and loaded on FEI Tecnai G2 Spirit BioTwin microscope (120 keV) for imaging. Cryo-EM grids (QUANTIFOIL, Germany) were plasma cleaned using PELCO easiGlow Glow Discharge System (Ted Pella, Redding CA). Aliquots of 3.5 µL of the purified virus sample (1.4 mg/mL) were applied to the grids, plotted for 2 s, and then plunge-frozen into liquid ethane using a vitrification robot (Vitrobot, Thermo Fisher). Grids were stored in liquid nitrogen until the date of screening and data collection. Data were acquired on a The Pennsylvania State University Huck Institutes Titan Krios electron microscope (300 keV) equipped with a spherical aberration corrector and Falcon 3EC direct detection camera. The microscope software EPU (*E pluribus unum*) was used to acquire data at a nominal magnification of x59,000 and an internal pixel size of 1.11 Å/pixel. A total of 1839 micrographs were recorded as movies (stacks of 54 frames) at an exposure rate of 0.89 e/Å^2^/frame and a total exposure time of 90.84 s. The nominal defocus range of −1.2 to −3.0 µm was applied during data collection.

### Image processing

Image analysis was performed using the cryoSPARC software package (v2.15.0)^43^. Aligned movie stacks were generated from raw micrographs after correcting for stage drift and anisotropic motion using patch motion correction. The output movies from motion correction were cropped to half the resolution in Fourier space (F-crop factor = ½). Parameters of the contrast transfer function (CTF) were estimated for each aligned movie in patch mode. Templates generated based on the 2D classification of 145 manually selected particles were used to pick 94,600 particles from 1835 micrographs. Further cleaning of the data using 2D classification and heterogenous refinement (3D classification) resulted in 5696 particles for subsequent refinement with higher-order CTF terms enabled (including beam-tilt, spherical aberration, trefoil, and tetrafoil) and icosahedral symmetry (I1) enforced. A final map at 8.3 Å resolution was obtained.

### Modeling

A homology model of ZIKV heterodimer prM-E was built using the Spondweni virus (SPOV) immature structure (PDB ID 6ZQI) as a template in MODELLER v9.25^44^. This software was used to model the predicted M-linker region. Ten models were constructed and evaluated by the Discrete Optimized Protein Energy (DOPE) scoring function, implemented in MODELLER v9.25, to select the best-scoring model. The resulting homology model with predicted M-linker was used to build an icosahedral asymmetric unit (ASU) consisting of three copies of prM/E heterodimer in ChimeraX. The generated ASU model of immature ZIKV was fitted into the 8.3 Å resolution map in ChimeraX. To further improve the fitting in the cryo-EM map, we used real-space refinement in Phenix. The chosen model was fitted in the map, and in rounds of refinement and manual adjustment of the loop connection, a final model was obtained. The quality of the modeled loop in the density was visually inspected in ChimeraX, and no clash between symmetry-related loops was observed. The full icosahedral map was cropped around the ASU within 8 Å distance using the “volume zone” command in ChimeraX; the cropped map, along with the prM/E heterodimers of the ASU, were the input for the real-space refinement in Phenix^45^. Graphical representations of maps and figures were generated in UCSF ChimeraX^46^, and for the prM/E figure, a 3 Å mask was applied to isolate the area of interest.

### Multiple sequence alignment

Amino acid sequences of flaviviruses were obtained from the NCBI Protein database, and multiple sequence alignments were performed using Clustal Omega. Alignment files were visualized and labeled using Jalview^47^. Sanger sequencing files were visualized using Serial Cloner (Serial Basics) and SnapGene software (Insightful Science; available at snapgene.com).

### Construction of mutant ZIKV cDNA clones

Mutant ZIKVs were generated on cDNA constructs of ZIKV Uganda strain MR766 under CMV promoter containing fluorescent protein Venus, mEmerald, or without a fluorescent tag^23,48^. Amino acid substitutions were introduced into the prM and E genes by site-directed mutagenesis using Phusion DNA polymerase (NEB, E0553S) and oligonucleotide primers (Integrated DNA Technologies, Inc.) (Supplementary Table 1). The PCR products were treated with *Dpn*I (New England BioLabs Inc., R0176L) and transformed into NEB Stable Competent *E. coli* (New England BioLabs Inc., C3040H). Plasmids were extracted using the Qiagen miniprep kit (Qiagen, 27104) and Qiagen midiprep kit (Qiagen, 12143) according to the manufacturer’s instructions. The sequences of the resulting plasmids were confirmed by Sanger sequencing.

### Transfection of cDNA clones

HEK 293 T cells or Huh 7.5 cells were grown in Opti-MEM I Reduced Serum Medium (Gibco, 11058-021) and were transfected with ZIKV cDNA clones using PEI MAX (Polysciences, 24765-1) or Lipofectamine 2000 (Thermo Fisher Scientific, 11668019), respectively. Transfected cells were incubated at 37°C in the presence of 5% CO_2_ for 12 h, and media were replaced with DMEM supplemented with 10% FBS, NEAA, and PS. Cell culture supernatants containing viruses were collected 4 days post-transfection and stored at −80 °C. Aliquots of virus stocks collected from cell-culture supernatants of transfected cells were used to infect Vero E6 monolayers to generate passage 1 (P1) stocks. After 5 days of incubation at 37 °C in the presence of 5% CO_2_, cell-culture supernatants containing the released virus were collected and stored at −80 °C.

### Fluorescence dilution assay

Virus stocks of WT and mutant ZIKV expressing Venus or mEmerald reporters were serially diluted in phosphate buffered saline (PBS) supplemented with 1% FBS and 1 mM each of CaCl_2_ and MgCl_2_ and added to monolayers of Vero E6 cells grown to confluency in 96-well plates (Greiner bio-one, 655180). Plates were incubated at 37°C in the presence of 5% CO_2_ for 4 days, and fluorescent cell clusters were counted under a fluorescent microscope.

### Plaque assay

Vero E6 cells were grown to confluent monolayers in 24-well plates (Greiner bio-one, 662160), and virus stocks were serially diluted in Minimum Essential Medium (MEM, GIBCO, 41500-018) supplemented with 1% FBS and 10 mM HEPES buffer, pH 7.5 (Sigma, H0887) were added to the cells. After a 1-h incubation at room temperature with rocking, wells were overlaid with DMEM containing 1% cellulose (Millipore Sigma, 435244) and 2% FBS. The plates were incubated for 6 days at 37 °C in the presence of 5% CO_2_. The cells were fixed with a mixture of 5% formaldehyde and 1% methanol (v/v in water) for 1 h, washed with PBS once, and stained with 0.1% Crystal Violet (Millipore Sigma, V5265) prepared in 20% ethanol. After 15 min, the stain was removed, and the cells were washed with water to visualize the plaques. Plaques were counted, and the results were plotted using GraphPad Prism 9.0 software.

### Growth kinetic analysis

Vero E6 cells grown to confluency in 6-well (Greiner bio-one, 657160) plates were infected in triplicate with virus stocks at a multiplicity of infection (MOI) of 0.1 or 0.01 and incubated for 1 h at 37 °C in the presence of 5% CO_2_. Infected cells were washed with DMEM, and media were replaced with DMEM supplemented with 10% FBS, NEAA, and PS and incubated at 37 °C in the presence of 5% CO_2_. Aliquots of 50 µl culture supernatant were collected and replaced with equal volumes of fresh media every 24 h post-infection, up to 96 h, and virus titers were determined by plaque assay.

### Quantification of virus release by quantitative RT-PCR (qRT-PCR)

HEK 293 T cells grown in 250 ml cell culture flasks (Greiner bio-one, 658175) were transfected with 10 µg of plasmid encoding WT or mutant ZIKV cDNA clones using PEI MAX. At 12 h post-transfection, the cell culture media were replaced with DMEM containing 2% FBS, NEAA, and PS and incubated at 37 °C in the presence of 5% CO_2_. At 4 days post-transfection, the cell culture supernatants were collected and clarified by centrifugation at 3214 × *g* for 15 min. Subsequently, the viruses were pelleted by ultracentrifugation at 175,000 × *g* for 90 min over a cushion of 25% sucrose in TNE buffer. The pellet containing the virus was resuspended in PBS, and RNA was extracted from samples using the Quick-RNA MiniPrep kit (Zymo Research, R1055) according to the manufacturer’s instructions. A QuantStudio 3 Real-Time PCR System (Applied BioSystems) using the Power SYBR Green RNA-to-CT 1-Step Kit (Thermo Fisher Scientific, 4389986) and primers -Forward TTGGTCATGATACTGCTGATTGC and -Reverse CCCTCCACGAAGTCTCTATTGC^49^ were used for qRT-PCR. The number of RNA molecules was estimated from a cT standard curve generated with the same primers and known molar concentration of ZIKV genomic RNA. Graphs were plotted and statistically analyzed using GraphPad Prism 9.0 software.

### Immunofluorescence assay (IFA)

Huh 7.5 cells grown on glass coverslips were transfected with ZIKV cDNA clones as described previously. Culture media were replaced with DMEM containing 2% FBS, NEAA, and PS 12 h post-transfection. Cells were fixed at 36 h post-transfection with 3.7% paraformaldehyde in PBS for 15 min and permeabilized with 0.1% Triton X-100 in PBS for 5 min at room temperature. Cells were blocked with 10 mg/mL bovine serum albumin (BSA, Sigma-Aldrich, A7906) in PBS overnight at 4 °C. Subsequently, the blocking buffer was removed, and cells were treated with primary antibodies for 2 h. The primary monoclonal mouse antibodies used were flavivirus-specific anti-E (a kind gift from Theodore C. Pearson) and anti-Giantin (Abcam, ab37266). The primary polyclonal rabbit antibodies used were ZIKV-specific anti-C (GeneTex, GTX134186) and anti-Giantin (Abcam, ab80864). The cells were washed 3 times with PBS, then treated with secondary antibody, fluorescein isothiocyanate (FITC) or tetramethyl rhodamine isothiocyanate (TRITC)-conjugated goat anti-rabbit and goat anti-mouse antibodies (Thermo Fisher Scientific, 31569 and T-2769) for 2 h. Nuclei were stained using Hoechst stain (Invitrogen, H3570) according to the manufacturer’s instructions, and then cells were washed 3 times with PBS. Coverslips were mounted onto microscope slides with FluorSave Reagent (Calbiochem, 3457). Images were acquired using a Nikon A1R confocal microscope fitted with a 60x oil objective lens at 1.4 numerical aperture (NA) and processed using the NIS Elements software (Nikon). Brightness and contrast were adjusted using Look Up Tables (LUT).

### Western blot analysis

HEK 293T cells were grown in 150 mm plates (Fisherbrand, FB012925) and transfected with 20 µg of each plasmid encoding WT or mutant ZIKV cDNAs using PEI MAX as described previously. Culture media was replaced with DMEM containing 2% FBS, NEAA, and PS 12 h post-transfection. At 72 h post-transfection, the cell culture supernatants were collected and centrifuged at 3214 × *g* for 15 min. Viruses were pelleted by ultracentrifugation at 175,000 × *g* for 90 min over a cushion of 25% sucrose in TNE buffer (25 mM Tris pH 7.4, 100 mM NaCl, 0.5 mM EDTA). Virus pellets were resuspended in 50 µl of RIPA buffer (50 mM Tris pH 8, 150 mM NaCl, 1% NP-40, 0.5% sodium deoxycholate, 0.1% sodium dodecyl sulfate), mixed, and incubated at 4 °C for 30 min. Virus samples were denatured, separated on a 12.5% SDS PAGE, and blotted onto a PVDF membrane. The membrane was blocked using 5% milk powder in TTBS (10 mM Tris pH 8, 120 mM NaCl, 0.01% Tween 20) for 1 h at room temperature. After washing 3 times in TTBS, the blots were probed with primary rabbit polyclonal ZIKV-specific anti-C antibody (GeneTex, GTX134186) for 12 h at 4 °C. The blots were washed three times with TTBS, then probed with HRP conjugated anti-rabbit secondary antibody (Sigma, A0545) and developed using the SuperSignal West Pico PLUS Chemiluminescent Substrate Kit (Thermo Scientific, 34577). Images were obtained using the Bio-Rad ChemiDoc XRS+ System.

## Results

### Cryo-EM structure of immature ZIKV

**Fig. 1:**
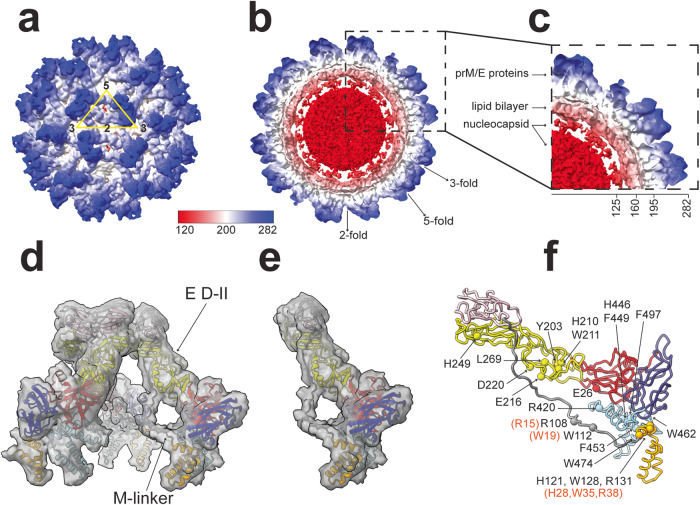
Cryo-EM map and homology model of immature ZIKV. **a** Icosahedrally averaged EM-density map rendered as surface. The asymmetric unit is indicated by a yellow triangle, and the two-, three-, and five-fold symmetry axes are labeled by numbers. **b** Cross-section and (**c**) quarter of a central slice of the map are shown; the maps in **a**–**c** are colored radially according to the given key. The labeled arrows on the cross-section indicate the icosahedral symmetry vertices. The densities corresponding to RNA at the nucleocapsid core (red), lipid bilayer (pink), and the prM/E heterodimers (blue) that form spikes of the immature capsid are labeled on the sliced map. Approximate distances (Å) from the center of the nucleocapsid core are indicated. **d** Part of the density map, rendered as a transparent surface (at a contour level of 2σ), carved around a docked homology model of three prM/E heterodimer proteins (constituting a single spike) from ZIKV, showing the quality of fitting. **e** Same as (**d**) but only shows the density and model of a single prM/E heterodimer. **f** The model of prM/E heterodimer is depicted as a cartoon; mutations of E and prM proteins discussed in the text are mapped onto the prM/E model and displayed as spheres. The model of the immature conformation of E protein consists of residues (1-504); the domains of E protein are indicated by different colors: domain I (red), domain II (yellow), domain III (blue), transmembrane helices (light blue). The model of prM protein consists of residues (1-168), including the pr domain (pink), the M domain (orange), and the linker (gray). The numbering of mutations in the prM protein (W19, H28, W35, and R38) shown in parenthesis is based on the sequence of the cleaved M protein. A 3 Å mask was used to isolate the density in to clearly visualize the asymmetric unit and the heterodimer (d and e).

Vero E6 cells treated with ammonium chloride were used to capture immature ZIKV MR-766 particles, which were purified from the culture supernatant. After vitrification, a homogeneous population of spiky particles was selected from micrographs for cryo-EM reconstruction resulting in an 8.3 Å resolution map of immature virus (Fig. 1), (Supplementary Figs. 1, 2), and (Supplementary Table 2). The icosahedrally averaged cryo-EM map revealed virus particles approximately 56 nm in diameter, decorated with spiky prM/E proteins, a characteristic feature of the immature conformation of ZIKV (Fig. 1a). Cross section of the density map clearly showed the outer glycoprotein layer of prM/E proteins; a lipid bilayer was observed underneath the spiky trimeric proteins. Furthermore, a noisy density presumably corresponding to the C protein was seen connecting the density of the nucleocapsid core to the lipid bilayer (Fig. 1b, c). The structure is similar in overall topology to the 9 Å and 9.8 Å structures of ZIKV purified from mosquito cells^19,20^. Visual inspection of the density map in ChimeraX by varying the contour levels (Fig. S2) revealed a density connecting pr and M domains that were consistent with the linker region, aa 86-119, (Fig. 1d–f). The densities of the M linker and E-DII run parallel without direct interactions.

The density of the linker region appeared weaker than that of portions of the pr and M domains, suggesting flexibility. Thus, the modest 8.3 Å resolution of our immature ZIKV map allowed modeling of the prM density and the N-terminal M linker region (Fig. 1d). A homology model of the immature conformation of ZIKV, containing E protein (aa 1-504) and uncleaved prM protein (aa 1-168) was generated based on the structure of SPOV (PDB 6ZQI) using Modeller^44^ (Fig. 1d). We further built the C alpha model for the prM into the density using the immature ZIKV structure (EMD-41037) which predicted the positions of M and E residues known to interact in the mature ZIKV structures (Supplementary Fig. 1), (Table 1). Real-space refinement (see Methods section) revealed the absence of clashes among residues within the modeled linker regions of prM/E heterodimers present in symmetry-related copies.

**Table. 1:** ZIKV M-E interactions predicted from mature cryo-EM structure (PDB ID 6CO8).

E-M interactions in the mature virus			
Residue 1	Residues 2 and 3, respectively	Type of interaction	Distance (Å) between residues 2 and 3, respectively
M H7	E H214*	Polar	3.0
M R10	E E216*	Polar	2.6
M R15	E E26*	Polar	3.1
M W19	E L269*	Hydrophobic	3.7
M R23	E E244	Polar	3.0
M E24	E S461*	Polar	3.9
M Y25	E W462*	Hydrophobic	3.6
M H28	E F463*	Hydrophobic	3.8
M W35	E G459	Hydrophobic	3.7
M R38	E D220	Polar	2.9
E E26	M R15*	Polar	3.1
E R73	^¤^	-	-
E K93	^¤^	-	-
E Y203	^¤^	-	-
E H210	^¤^	-	-
E W211	M W19*	Hydrophobic	3.5
E H214	M H7*, M R10*	Polar	3.0, 4.0
E E216	M R38, M R10*	Polar	3.8, 2.8
E D220	M N34, M R38	Polar	3.1, 2.9
E E244	M S22, M R23	Polar	2.7, 3.0
E H249	M S16	Polar	3.5
E H266	M W19*	Hydrophobic	4.0
E L269	M W19*	Hydrophobic	3.7
E E274	M W19*	Polar	3.7
E R420	M R15*	Polar	3.7
E F449	^¤^	-	-
E W462	M Y25*	Hydrophobic	3.7

*Interaction between two M/E heterodimers

^¤^E side chain interaction with prM.

### The structure-based mutational analysis identifies prM and E amino acid interactions critical for ZIKV growth

**Fig. 2:**
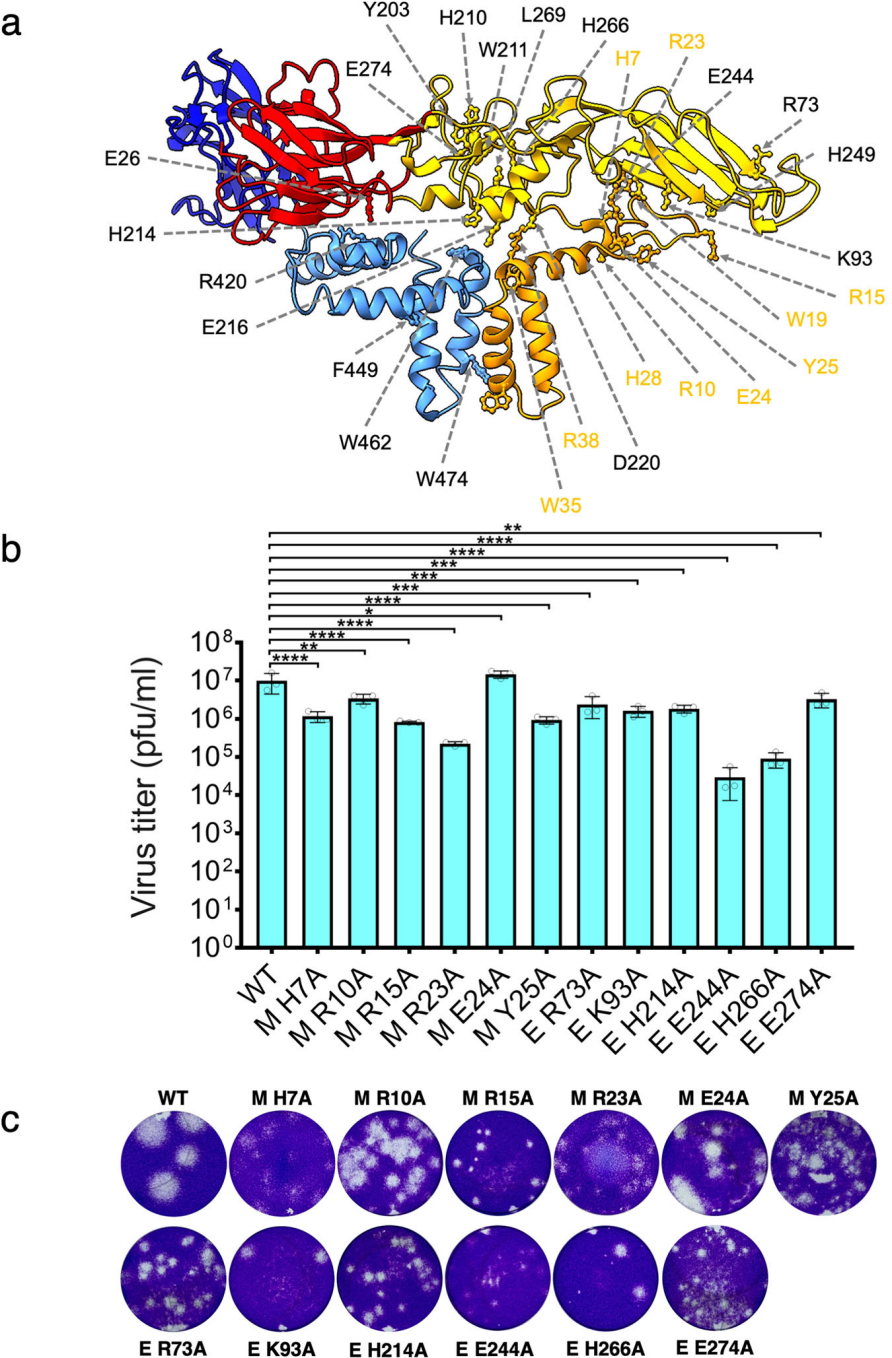
Mutational analysis of prM and E identifies amino acid residues critical for ZIKV growth. **a** Structure of mature ZIKV M and E proteins (PDB: C6O8) with amino acid residues selected for mutagenesis shown in ball and stick and labeled. The M residues are labeled in orange. Protein domains are indicated as follows: M orange, E D-I red, E D-II yellow, E-DIII blue, and E TM helices light blue. **b** Graph showing the titers of WT ZIKV and the plaque-forming mutants estimated by plaque assays. Data represent the mean of three replicates of a single biological experiment with standard error of the mean (SEM). Significance levels were calculated using one-way ANOVA followed by Dunnett’s multiple comparisons test (****p < 0.0001). **c** Representative images showing plaque morphology of WT ZIKV and plaque-forming mutants on confluent monolayers of Vero E6 stained with crystal violet.

We predicted the possible interacting residues between M and E proteins in mature ZIKV from the high-resolution structures (PDB: 7JYI) and (PDB: 6CO8)^23,24^. We identified 27 amino acids, specifically, H7, R10, R15, W19, R23, E24, Y25, H28, W35, R38, from M protein and E26, R73, K93, Y203, H210, W211, H214, E216, D220, E244, H249, H266, L269, E274, R420, F449, (Supplementary Fig. 1), and W462 from E protein for further alanine scanning mutational analysis (Fig. 2a), (Supplementary Fig. 3), (Supplementary Fig. 4), and (Table 1). We also included previously characterized assembly mutation E W474A and furin cleavage site double mutation prM R92A, R93A in our experiments^23,50^. Mutations were introduced into the WT ZIKV cDNA clone and WT ZIKV cDNA clone expressing mEmerald by site-directed mutagenesis, and WT and mutant viruses were generated after transfecting the resulting cDNA clones into HEK 293 T cells. The effect of alanine substitution on virus release was tested by fluorescence dilution assays of cell culture supernatants of transfected cells (P0) on Vero E6 monolayers. The virus titers were determined by standard plaque assays of cell culture supernatants of infected cells (P1) on Vero E6 monolayers (Table 2).

**Table 2:** Plaque phenotypes of Zika viruses used in this study.

Protein	Location	Residue	Plaque phenotype (NP, SP, MP,& LP)
*prM*	*pr*	*R92, R93*	*NP*
M	N-term	H7	MP
M	N-term	R10	MP
M	N-term	R15	SP
M	N-term	W19	NP
M	H1	R23	SP
M	H1	E24	LP
M	H1	Y25	MP
M	H1	H28	NP
M	H1	W35	NP
M	H1	R38	NP
E	DI	E26	NP
E	DII	R73	MP
E	DII	K93	MP
E	DII	Y203	NP
E	DII	H210	NP
E	DII	W211	NP
E	DII	H214	MP
E	DII	E216	NP
E	DII	D220	NP
E	DII	E244	SP
E	DII	H249	NP
E	DII	H266	MP
E	DII	L269	NP
E	DII	E274	SP
E	H1	R420	NP
E	H1	F449	NP
E	TM1	W462	NP
*E*	*TM1*	*W474*	*NP*
*-*	*-*	*WT*	*LP*

A total of 27 alanine substitution mutations were made on cDNA clones of ZIKV. Plaque phenotypes of Vero E6 cells were determined by plaque assay and crystal violet stain. Protein, domain, residue, and plaque size are indicated where the plaque size characterizations are NP (no plaque), S (1–2 mm plaque), M (2–3 mm plaque), and L (4–5 mm plaque). The WT, maturation defective control (pr R92A, R93A), and defective assembly control (E W474A) are included in the table and italicized. Plaque morphologies and titers of the mutant and WT viruses were determined after fixing and staining plaque assays 6 days post-infection (Fig. 2b, c) and (Table 2). The prM and E mutations resulted in lethal phenotypes with no plaques, as well as small (1–2 mm), medium (2–3 mm), and large (4–5 mm) plaque phenotypes. The wild-type (WT) virus and M mutation, E24A, formed large plaques with a similar average titer. M mutations H7A, R10A, and Y25A resulted in medium plaques with approximately 1-log or less difference in titer from WT, suggesting these amino acids are not critical for virus assembly and release. M mutations R15A and R23A produced small plaque phenotypes with approximately a 2-log reduction in virus titers compared to WT. Finally, M mutations W19A, H28A, W35A, and R38A, as well as the prM R92A and R93A maturation mutant, were lethal, resulting in no plaque phenotype. Akin to the M protein mutations, the E protein mutations R73A, K93A, and H214A produced medium plaque morphologies compared to the WT virus with an approximately 1-log reduction in virus titer compared to the WT virus. The E protein H266A mutation showed a medium plaque phenotype with a 2-log reduction from the WT virus titer. In contrast, E protein E E274A mutation showed a small plaque phenotype with approximately a 1-log reduction from the WT virus titer. E protein mutation E244A was the most attenuated plaque-forming mutant virus with a small plaque phenotype and greater than 2-log reduction in viral titer compared to the WT virus. Overall, for mutations that resulted in small and medium plaque phenotypes, we inferred that these residues play essential roles in the flavivirus lifecycle; however, they are not critical for virus assembly and maturation. Lastly, E protein mutations E26A, Y203A, H210A, W211A, E216A, D220A, H249A, L269A, R420A, F449A, and W462A, as well as the assembly mutant E W474A, were lethal, resulting in no plaque phenotype. Therefore, these amino acids are defective in critical steps in virus assembly or maturation.

### Growth kinetic analysis of plaque-forming prM and E mutants identify mutations affecting virus release

**Fig. 3:**
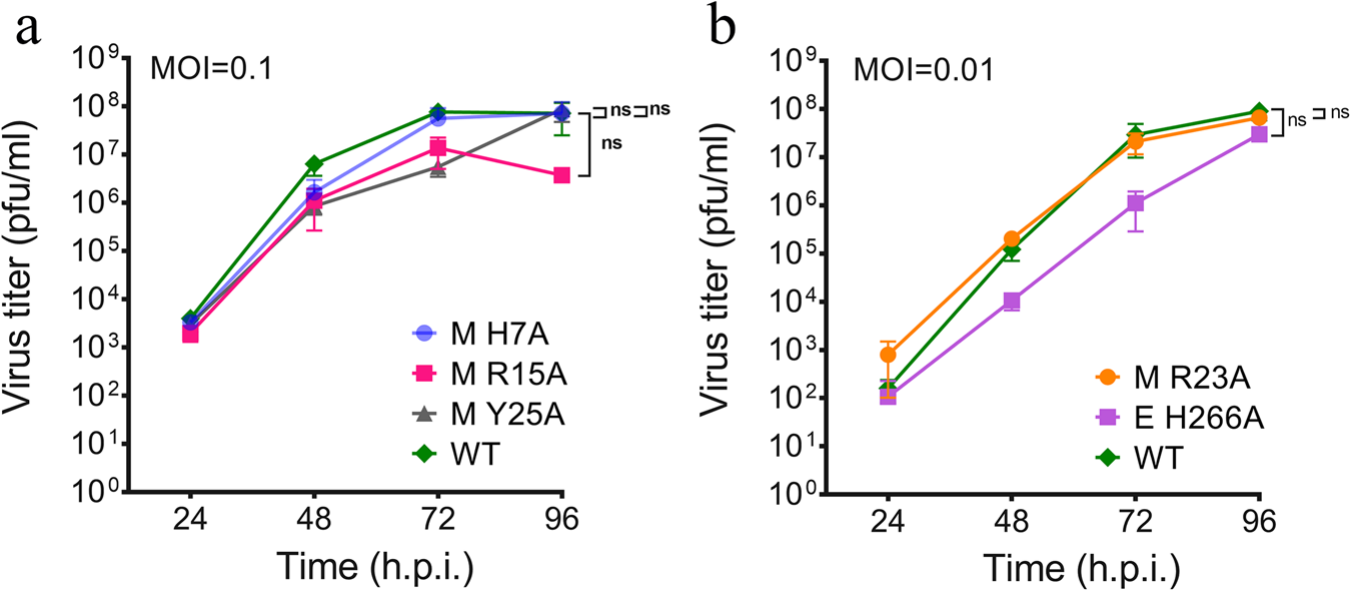
Growth kinetic analysis of plaque-forming prM and E mutants. Vero E6 cells were infected with WT and mutant viruses at an MOI of 0.1 (**a**) or 0.01 (**b**). Cell culture supernatants were harvested every 24 h up to 96 h, and virus titers were determined by plaque assay. Data represent the mean of three biological replicates with SEM. Significance levels of the curves were calculated using one-way ANOVA followed by Dunnett’s multiple comparisons test (n.s. not significant).

We next determined the effect of mutations on the growth kinetics of plaque-forming prM and E mutant viruses that showed reduced plaque sizes and viral titers compared to WT virus (Fig. 2b, c) and (Table 2), which included M H7A, M R15A, M R23A, M Y25A, and E H266A mutants. For M H7A, M R15A, and M Y25A, virus stocks of sufficiently high viral titers were obtained to infect the cells with an MOI of 0.1 (Fig. 3a). Due to the low titers for M R23A and E H266A, we infected the cells at an MOI of 0.01 (Fig. 3b). Growth kinetic analysis shows ZIKV mutants M H7A, M R23A, and M Y25A maintained a growth rate close to that of WT. However, E H266A and M R15A exhibited markedly reduced growth kinetics compared to the WT virus. At 24 h post-infection (hpi), E H266A grew slower than WT, with virus titer reduced by approximately 2-log, and continued to grow slowly at 48 and 72 hpi. By 96 hpi, viral titers for E H266A reached near WT levels (Fig. 3b). The M R15A mutant grew slowly throughout. At 96 hpi, the titer was approximately 2-log lower than the WT virus (Fig. 3a). Our growth kinetic data indicate that amino acids E H266 and M R15 participate in the virus life cycle. Still, they are not critical for the assembly or release of infectious virus particles.

### Determining the assembly or maturation defects of the non-plaque-forming prM and E mutants

**Fig. 4:**
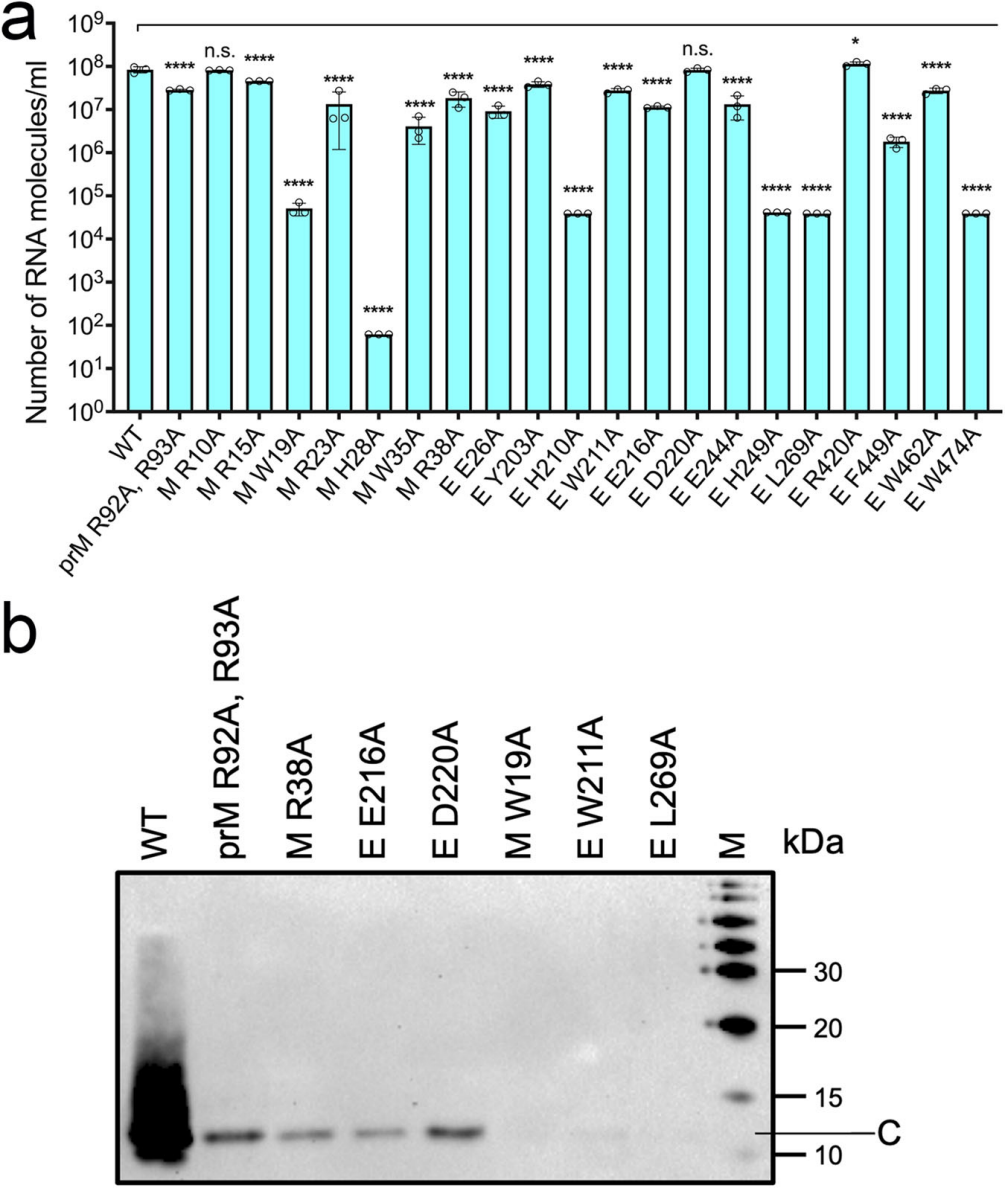
Analysis of non-plaque forming mutants for virus release. **a**, **b** Cell culture supernatants from HEK 293 T cells transfected with cDNAs of WT and mutants were pelletted by ultracentrifugation on a sucrose cushion and analyzed for the presence of RNA via qRT-PCR (**a**) and virus release via western blot analysis (**b**). **a** Graph showing the number of ZIKV RNA molecules/ml in the cell-culture supernatants. The number of RNA molecules was calculated using a standard curve derived from a known concentration of ZIKV genomic RNA and resulting Ct values. Data represent the mean of three replicate experiments with SEM. Data were normalized to the number of RNA extracted from mock-transfected cells. Significance levels were calculated using one-way ANOVA followed by Dunnett’s multiple comparisons test (****p < 0.0001, *p < 0.1, n.s. not significant). **b** Western blot showing the release of the virus into the cell culture supernatant. Pellets obtained after ultracentrifugation of the culture supernatants of HEK 293 T cells were separated on a 12.5% SDS PAGE and probed with antibodies that detect ZIKV capsid protein. The label M indicates the protein ladder, and the band corresponding to the capsid protein (12 kDa) is labeled C.

Based on the plaque assays, we have identified mutations in prM and E proteins of ZIKV that failed to produce infectious plaque-forming units (Table 2). The lethal phenotype could be due to assembly defects where no viruses are released or maturation defects where defective or immature viruses are released. To categorize the lethal phenotype mutants as either assembly or maturation mutants, first, we performed qRT-PCR to determine the number of viral RNA molecules released into the cell culture supernatant as assembled virus particles, thus indirectly estimating the number of virus particles released (Fig. 4a). For this experiment, we transfected HEK 293 T cells with 10 μg of DNA representing the cDNA of M protein mutants W19A, H28A, W35A, R38A and E protein mutants E26A, Y203A, H210A, W211A, E216A, D220A, H249A, L269A, R420A, F449A, W462A. We also included WT (large plaque), assembly mutant E W474A (no plaque), maturation mutant prM R92A, R93A (no plaque), and M mutants R10A (medium plaque), R15A (small plaque), R23A (small plaque), and E mutant E244A (small plaque) for comparison. Mock-transfected cells were used as RNA controls. Virus particles were pelleted from the clarified cell culture supernatants by ultracentrifugation on a sucrose cushion, and RNA was extracted from pellets. Our qRT-PCR results show the number of RNA molecules released by the maturation control prM R92A and R93A is comparable to the WT virus sample, at 2.84 × 10^7^ and 8.45 × 10^7^ RNA molecules/ml, respectively. In contrast, the number of RNA molecules released by the assembly mutant W474A was significantly reduced, at 3.88 × 10^4^ (Fig. 4a). Of all the mutants tested, M W19A, M H28A, E H210A, E H249A, and E L269A released 3-log less viral RNA per ml of culture supernatant compared to WT, matching E W474A assembly control, suggesting significantly reduced virus particles were released from cells possibly due to assembly defects. Mutants M R10A, E D220A, and E R420A released RNA molecules close to WT levels, suggesting assembly is unaffected by these mutations. Mutants M W35A, M R38A, E E26A, E Y203A, E W211A, E E216A, E E244A, E F449A, and E W462A released RNA levels relatively less than WT but close to the maturation control prM R92A, R93A, suggesting these small or medium plaques to estimate their defects in maturation or entry (Supplementary Table 3). mutants are also releasing particles that are unable to infect cells, therefore, we classify them as maturation mutants. We calculated the specific infectivity of severely affected mutants that form. The M mutants R10A, R15A, and R23A showed specific infectivity close to wild-type values, suggesting most of the particles released are infectious, but a reduced number of particles are released. In contrast, the E mutant E244A showed significantly affected specific infectivity, suggesting particles released have maturation or entry defects.

To corroborate the assembly or maturation defects of non-plaque forming mutants identified from the qRT-PCR analysis, we performed a western blot analysis of culture supernatant from HEK 293 T cells transfected with the corresponding cDNAs. For this experiment, we selected the candidate assembly mutants M W19A, E W211A, and E L269A and maturation mutants M R38A, E E216A, and E D220A. Cell culture supernatants pelleted by ultracentrifugation were separated on SDS-PAGE gel, blotted onto PVDF membrane, and probed for the presence of ZIKV capsid protein using ZIKV anti-capsid primary antibody. A band corresponding to the ZIKV capsid protein at 12 kDa was detected for M R38A, E E216A, and E D220A mutants and the maturation control prM R92A, R93A. However, a C protein band was not seen for M W19A, E L269A, and E W211A mutants (Fig. 4b). The immature mutants that showed significant RNA release, namely M R38A, E 216A, and E D220A, also showed release of virus particles as detected in western blot analysis confirming viral release into the supernatant. As expected, band intensity for these mutants was noticeably lower than that of the WT virus. WT ZIKV and plaque-forming mutant viruses can re-infect, increasing virus release and higher capsid band intensity. In contrast, maturation defective mutants are noninfectious, therefore, infection is limited to the viral particles produced upon transfection, leading to lower-intensity capsid bands. The low band intensity observed for the maturation-defective mutant viruses was comparable to the known maturation mutant prM R92A, R93A.

### Detection of defects in virus assembly and maturation of mutants by immunofluorescence analysis (IFA)

**Fig. 5:**
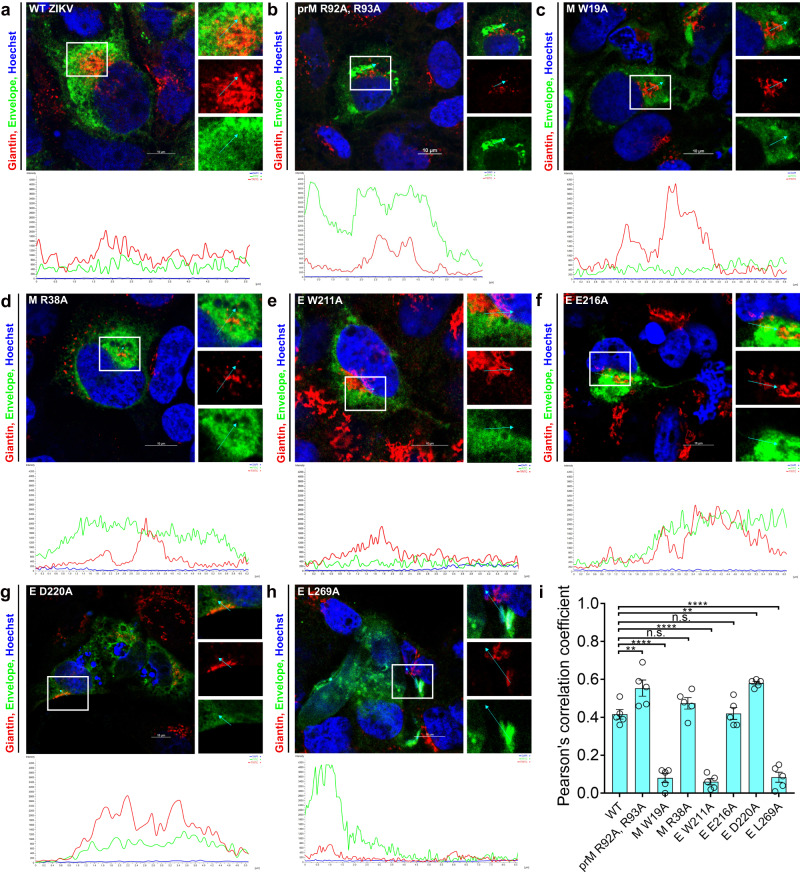
Immunofluorescence analysis for colocalization of E protein with Golgi. Confocal micrographs of IF assays to detect the colocalization of E protein to Golgi. Huh 7.5 cells were transfected with WT and mutant ZIKV cDNAs (**a**)WT ZIKV, (**b**) prM R92A, R93A, (**c**) M W19A, (**d**) M R38A, (**e**) E W211A, (**f**) E E216A, (**g**) E D220A, (**h**) E L269A, fixed after 36 h post-transfection, and probed using Golgi marker Giantin (red) and E (green) antibodies. Regions of interest are enlarged to the right and displayed as merged and separate channels. Analyzed regions are indicated with a cyan arrow, and corresponding profiles are displayed at the bottom, where distance is measured as μm and intensity is measured as arbitrary fluorescence units (AFU). **i** Pearson’s correlation coefficients to quantify the colocalization of E protein with Golgi marker Giantin. Data represent an average of 5 measurements with SEM. Significance levels were calculated using one-way ANOVA followed by Dunnett’s multiple comparisons test (****p < 0.0001, **p < 0.01, n.s. not significant).

**Fig. 6:**
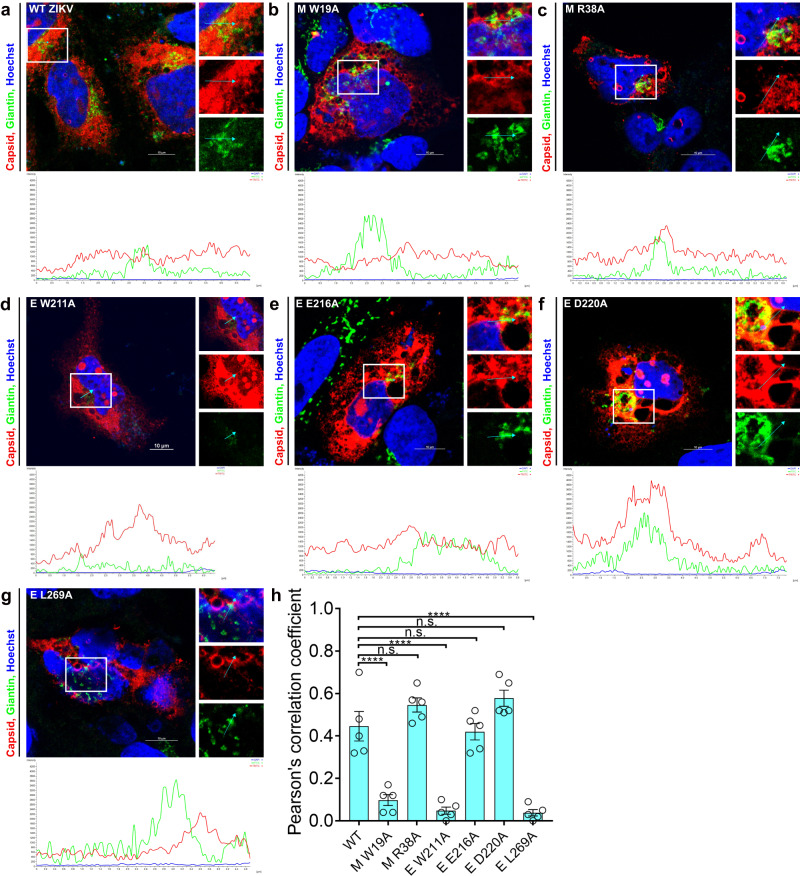
Immunofluorescence analysis for colocalization of C protein with Golgi. Confocal micrographs of IF assays to detect the colocalization of C protein to Golgi. Huh 7.5 cells were transfected with WT and mutant ZIKV cDNAs (**a**)WT ZIKV, (**b**) M W19A, (**c**) M R38A, (**d**) E W211A, (**e**) E E216A, (**f**) E D220A, (**g**) E L269A, fixed after 36 h post-transfection, and probed using capsid (red) and Golgi marker Giantin (green) antibodies. Regions of interest are enlarged to the right and displayed as merged and separate channels. Analyzed regions are indicated with a cyan arrow, and corresponding profiles are displayed at the bottom, where distance is measured as μm and intensity is measured as arbitrary fluorescence units (AFU). **h** Pearson’s correlation coefficients to quantify the colocalization of C protein with Golgi marker Giantin. Data represent an average of 5 measurements with SEM. Significance levels were calculated using one-way ANOVA followed by Dunnett’s multiple comparisons test (****p < 0.0001, n.s. not significant).

We further characterized the non-plaque forming prM and E mutants by IFA to detect the presence of capsid and E proteins in the Golgi that defines virus assembly. Mutations M W19A, M R38A, E W211A, E E216A, E D220A, and E L269A were introduced into the WT cDNA clone of ZIKV, without a fluorescent protein tag, along with the prM R92A, R93A maturation control, and E W474A assembly control mutations. The cDNA clones were transfected into Huh 7.5 cells, and after 36 h, the cells were fixed with 3.7% paraformaldehyde and probed with primary antibodies against ZIKV C and E proteins along with antibody detecting Golgi marker Giantin. In the cells transfected with WT and the prM R92A, R93A maturation control cDNAs, ZIKV E protein colocalizes to Giantin (Fig. 5a, b), indicating virus assembly and trafficking of structural proteins through the Golgi. Colocalization of E and C with Giantin were also observed in cells transfected with M R38A (Figs. 5d and 6c), E E216A (Figs. 5f and 6e), and E D220A (Figs. 5g and 6f) mutants, suggesting these mutant viruses could assemble and traffic through the Golgi. In contrast, no E-Giantin and C-Giantin colocalization could be detected in cells transfected with M W19A (Figs. 5c and 6b), E W211A (Figs. 5e and 6d) and E L269A (Figs. 5h and 6g). We quantified the colocalization between E and C with Giantin by calculating Pearson’s correlation coefficient from the confocal micrographs using Nikon Elements Software (Figs. 5i and 6h). For the Mutants M R38A, E E216A, and E D220A, C and E colocalized with Giantin with coefficient values similar to WT ZIKV. However, the mutants M W19A, E W211A, and E L269A had significantly lower coefficient values for C and E colocalization with Giantin. We also verified the expression of C and E proteins (Supplementary Fig. 5) in the transfected cells.

## Discussion

Here, we describe a new flavivirus latch and lock maturation model. The sequential latch and lock mechanism takes advantage of electrostatic and hydrophobic interactions between M and E proteins of ZIKV to mediate the maturation-related rearrangements and stabilize the metastable virion primed for subsequent infection. We determined a cryo-EM structure of immature ZIKV (Fig. 1), which resolved the density corresponding to the N-terminal M linker region connecting the pr domain and the TM helices. Based on the fitting of the model we generated using the high-resolution structure of SPOV prM, we identified that the critical interactions occurring in the mature structure are absent in the immature structure, suggesting that they form in the secretory pathway during maturation. Although several prM and E interactions have been identified as critical based on cryo-EM analyses, thus far, only a few of these have been experimentally validated. The strength of our study lies in the extensive molecular characterization of M and E interactions identified from immature and mature cryo-EM structures of ZIKV to identify critical residues that drive the maturation pathway.

Electrostatic interactions between prM and E have been identified previously, including M E31 interacting with E K93 and E H246, which is critical for VLP production in JEV^51^. Here, we have mapped crucial electrostatic interactions involving M R38 with E E216 and E D220 that are essential for ZIKV maturation (Supplementary Fig. 1). A second set of electrostatic interactions includes M R15 and E E26 from DI and E R420 from H1 that stabilize the smooth M-E heterodimers in the mature virus. The ZIKV residues M R15 and E E26 are akin to residues M K16 and E D28 (Supplementary Fig. 3) that form salt bridges and stabilize M and E heterodimers in Murray Valley encephalitis virus (MVEV) and KUNV^35^. In addition, we found that the lethal mutations M R38A, E E26A, Y203A, E W211A, E E216A, E D220A, and E R420A release viral RNA similar to the maturation control prM R92A R93A with furin a cleavage site mutation that releases only immature particles^50^. Therefore, the mutants that release virus particles comparable to WT levels, M R38A, E E26A, E E216A, E D220A, E, and R420A, are not defective in virus assembly, indicating that the salt bridge between conserved residues M R38A and E D220A and electrostatic interactions between E E26A, and R420A, are required for virus maturation. Western blot analysis of the purified virus samples using an anti-capsid antibody detected the presence of released virus particles in the maturation control prM R92A R93A and maturation mutants M R38A, E E216A, and E D220A. We also detected the localization of C and E proteins in the late Golgi using anti-C, anti-E, and anti-Giantin antibodies in the WT and mutants, including the maturation mutants that release virus particles determined from the qRT-PCR and western blot analysis. We detected a complete colocalization of C and E proteins akin to the WT virus in IF analysis for all the mutants that release virus particles (Supplementary Fig. 5). Taken together, the new critical electrostatic interactions of prM and E heterodimers between M R38A, E E216A, and E D220A that we describe here as “latching interactions” affect maturation and did not significantly affect virus assembly or release.

Our work also provides evidence for two sets of hydrophobic sidechain interactions; first involving M W19, with E W211, and E L269 from DII of the opposite E subunit interacting across the E-E dimer here, which we describe as “locking interactions” that affect virus release. Second, the M protein H1 residue W35 and E protein TM1 residue W462 interact with the outer layer of the lipid bilayer, stabilizing the helical regions of M and E proteins in the mature virus structure. Residues in the helical domain of DENV1 M protein have been previously described to modulate the prM cleavage, maturation of particles, and virus entry in a VLP system^52^. The ZIKV M W19A and E L269A mutants did not show detectable levels of release virus particles (Fig. 4B). In contrast, the E W211A mutant showed virus release in qRT-PCR but at a highly reduced level, compared to the maturation mutant prM R92A R93A that releases immature virus and the assembly mutant E W474A that is defective in virus release^23^. Notably, the locking interactions involving M W19 can only form after the furin cleavage of prM the N-terminal 20 residues of M are internalized to localize to the underside of E-E dimers at low pH assisted by the protonation of E His27 and His244 to rearrange below the E dimer^36^. The locking interactions are also strengthened by the M H28, which interacts with E F463. Given that the mutations hindering these hydrophobic interactions could affect the stability of virus particles, it could lead to the premature rising of E protein at low pH caused by the absence of locking interaction. Although the lethal mutations M W35A and E F449A showed virus release, albeit with a 2–3 log reduction in RNA copy numbers, indicating defects in inefficient assembly, maturation, or infectivity. The E protein lethal mutation Y203A showed virus release, suggesting that this residue is likely involved in virus receptor binding and entry. The E W462 residue interacts with M Y25 from the perimembrane helix H1 at the M-E heterodimer interface, whereas E W211 interacts with M W19 from the neighboring M-E heterodimer. We hypothesize that the hydrophobic interactions of E W462 and M Y25 are essential for the formation and stability of smooth immature virus particles at low pH, a prerequisite for the furin cleavage of prM. In IF analysis, reduced Golgi localization was identified for assembly mutants M W19A, E W 211 A, and E L269A. Thus, the locking mutants likely reach the Golgi and undergo low pH-mediated rearrangement and furin cleavage but are defective in virus release due to the instability of E protein dimers caused by the absence of locking interactions.

**Fig. 7:**
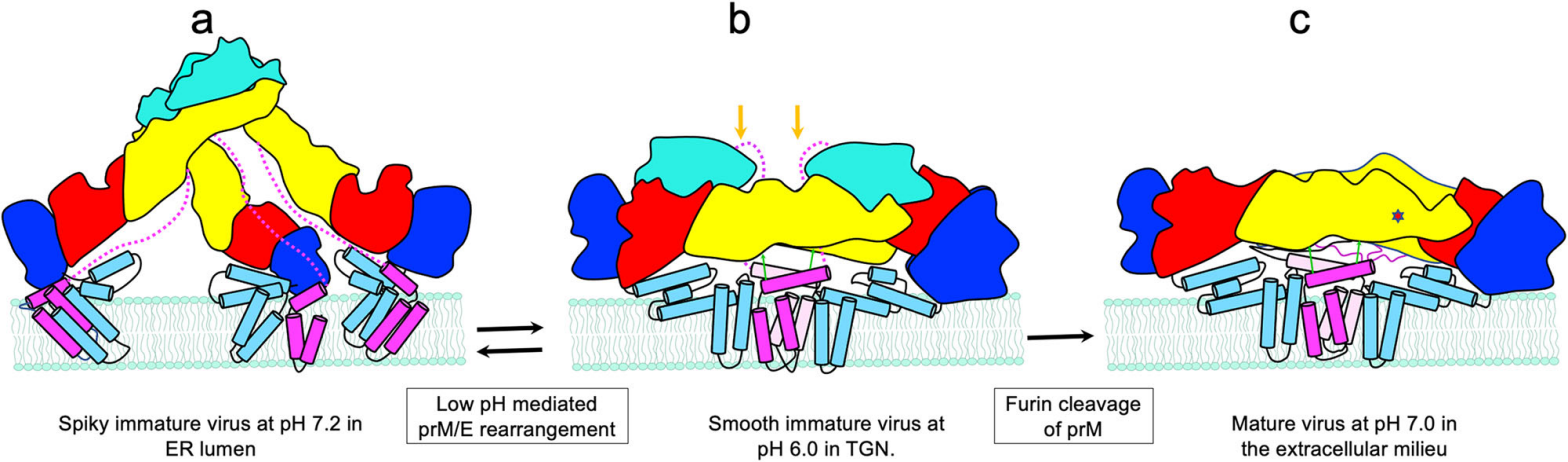
Latch and lock model for ZIKV maturation. **a**–**c** ZIKV maturation stages and sequence of events highlight the role of “latch and lock” interactions in the pH-mediated rearrangements of prM/E and M/E interactions in stabilizing the mature virus. Organization of prM/E proteins on the viral membrane of the spiky immature (**a**), smooth immature (**b**), and M and E proteins on the mature virus (**c**) during maturation. The cartoons are based on the cryo-EM structure of immature ZIKV (EMD-41037, this study) crystal structure of a smooth immature TBEV virus (PDB 7QRE), and the proposed model from immature SPOV structure (PDB 6ZQW) and mature ZIKV (PDB 6CO8). The E protein domains are represented as DI: red, DII: yellow, DIII: dark blue, and the helical region: light blue. The prM domains are represented as pr: teal and M: magenta. **a** Immature particles in the ER have spiky trimeric prM/E heterodimers at neutral pH. A dotted line in magenta indicates the putative orientation of the ZIKV M protein based on the SPOV model. **b** When the immature virus is transported through TGN, the low pH induces a conformational change triggered by the protonation of histidine residues and E protein hinge movement, leading to a smooth immature virus. The pr is wedged at the E-E dimer interface, attached to E DII, protecting the fusion loop on one side and making inter-protomer interactions with DI and DIII on the other. The stability of the smooth immature virus, which allows the exposure of the furin cleavage site, requires electrostatic latching interactions involving residues M R38, E E216, and E D220, indicated by green arrows. Orange arrows indicate furin cleavage sites. **c** After the furin cleavage that releases the M protein from pr bound to E, the N-terminus of the M protein slips through a hole in the E protein facilitated by protonation of histidine residues opening a hinged lid near glycan loop 150. The N-terminal 20 residues of M protein form an extended loop and localizes under the E protein dimers, which allows the formation of stabilizing locking interactions. Hydrophobic inter-heterodimer M-E interactions, including M W19, E W 211, and E L269, lock the dimeric E structure from rising at low pH, preventing the premature exposure of E protein after prM cleavage. The metastable mature virus is poised to start infection and fusion during entry. A red star indicates E-M locking interactions, including M W19, E W 211, and E L269, hidden between two E dimers.

Combining the structural analysis and experimental validation of residues involved in the prM/E and M/E ectodomain interaction, we propose a new “latch and lock” model for ZIKV maturation (Fig. 7). In the immature trimeric spike form, the pr covers the fusion loop in E and prevents fusion with host cell membranes^21^. The protonation of conserved histidine residues in prM and E, facilitated by the flexibility of hinge regions of E protein, triggers the initial low pH-mediated conformational switch of prM and E^20,53–55^. We found that ZIKV histidine residues M H28, E H210, and E H249 are critical for the low pH mediated rearrangement of prM and E as reported for M H39 and E H244 of DENV^56,57^. Even though the protonation of prM linker His101 is predicted to promote the prM-E dissociation and release of E in SPOV, our results show that prM residue His 101, which becomes residue 7 in M after furin cleavage of prM (Supplementary Fig. 3) is not critical for ZIKV release^18^. Furthermore, the pr domain binding to the E DII remains unchanged in the spiky immature structure at neutral pH and smooth immature virus at low pH. Stabilizing interactions between pr and E molecules at the tip of the spike and N-terminal region of M with E residues 209-215 and 258-265 have been detected from a 6 Å cryo-EM map of immature DENV 1^53^. In addition, the acidic pH X-ray structure of TBEV pr/E dimer has revealed that pr stays attached to E at acidic pH due to electrostatic complementarity, and a hinged-lid of the glycosylation 150-loop opens allows pr-binding in a pocket to generate smooth immature particles^32^. Collectively, our results indicate that instead of the structural rearrangements predicted to be induced by prM protein, the reorganization driven by the pH-mediated hinge movement of E protein with the pr domain bound to E-DII leads to the reversible, spiky to smooth transition of prM/E in the immature virus. Thus, we demonstrate that the smooth conformation allows the formation of latching interactions, including M R38A, E E216A, and E D220A, between the positively charged and negatively charged prM and E residues, allowing the formation of salt bridges at low pH. The E protein domain movements also result in E interdomain interaction, including a salt bridge between E E26 and E R420, providing stability to the smooth immature structure, thereby allowing the exposure of the furin cleavage site and subsequent cleavage of prM. The protonation of E protein histidine residues allows the irreversible rearrangement of the cleaved N-terminal domain of M protein to internalize and form hydrophobic locking interactions between residues M W19A and E L269A underneath the E protein dimers. Taken together, stabilizing the “raft” interactions between M/E heterodimers results in a virion with 90 E protein dimers organized in a herringbone pattern in a metastable conformation.

Along with mature particles, flavivirus-infected cells release prM-containing immature and partially mature mosaic particles, representing an intermediate subpopulation in the virus maturation pathway^23,50,58,59^. The prM-containing, partially mature infectious virions have been shown to contribute to pathogenesis during primary DENV infections^60^. Indeed, the cryo-electron tomography analysis of DENV-2 mosaic particles has provided evidence for the maturation-related reorganization of glycoproteins initiated from one or more nucleation centers^61^. In contrast, it has been proposed that the simultaneous rearrangements of multiple trimeric spikes from neighboring asymmetric units at low pH may lead to steric problems. Therefore, an alternate model involving simple glycoprotein translational movement was proposed to describe the immature to mature reorganization^18^. Furthermore, the prM/E structural transition was proposed to initiate at the fivefold axis, which can minimize the topological constraints, preventing clashes of prM/E spikes during maturation^17^. In comparison, our proposed maturation model suggests that prM/E reorganization of spiky to smooth immature virus transition at low pH occurs uniformly, with maturation initiating from the site of furin cleavage and progressively proceeding to the 180 prM locations in the immature virus, which might not be driven to completion in all immature virus particles. Given that the remaining uncleaved prM molecules are unable to lock the E protein from rising at neutral pH to a spiky structure, incomplete cleavage of prM leads to the production of immature and partially mature mosaic particles containing a combination of prM/E trimeric spikes and M/E dimeric rafts.

Enveloped viruses with pH-sensitive glycoproteins employ ion channels to mediate pH balance in the secretory pathway, such as influenza M2, HIV Vpu, HCV P7, and alphavirus 6K^62–64^. However, a viral ion channel protein is not present in flaviviruses to enable the pH balance of envelope glycoprotein, suggesting that the virus achieves the stability of E glycoprotein with a chaperon-like function of prM protein. Whereas the E protein bound to cleaved pr allows the mature flavivirus to pass through the acidic compartments of TGN without membrane fusion, the pr retention is not likely to prevent the inherent flexibility of E protein with its hinge movement-associated domain rearrangement, implicating the functional role of M protein in locking the E rising which is required for low pH fusion. The “latch and lock” mechanism we present here explains the interactions involved in the low pH-mediated translational movements as are necessary for the reversible transition from spiky immature trimer to smooth immature dimer and further irreversible change to smooth mature virion, ascribing a critical function of M protein in locking the E protein dimers from premature rising, thus protecting the mature virion from inactivation in the secretory pathway. The heterogeneity of the released particles can more definitively be determined at the molecular and phenotypic levels using mass spectrometry (MS) and cryo-EM^65^. It would, therefore, be pertinent to test the noninfectious and highly affected mutant viruses produced due to alterations in the maturation-mediated rearrangements in future cryo-EM and cryo-electron tomography studies. The structural information of mutant viruses on the degree of maturation and structural heterogeneity of noninfectious particles will provide a detailed understanding of flavivirus morphogenesis, which might pave the way to develop new interventional strategies against these reemerging human pathogens.

## Supplementary information

Supplementary information

## Data Availability

Cryo-EM electron density map of the native immature ZIKV has been deposited in the Electron Microscopy Data Bank, https://www.ebi.ac.uk/pdbe/emdb/ (accession number EMD-41037). The modeling data supporting this study’s findings are available from the corresponding author upon request. All other data generated during this study are included in this manuscript and its supplementary information files.
