# Supplementary material for: Zika virus M protein latches and locks the E protein from transitioning to an immature state after prM cleavage
Source: Npj Viruses. 2023 Nov 6;1:4. doi: 10.1038/s44298-023-00004-2 (PMC11721464; doi:10.1038/s44298-023-00004-2)
Supplement: Supplementary file 1 — Supplementary information [file 44298_2023_4_MOESM1_ESM.docx]

**SUPPLEMENTARY INFORMATION**

**Supplementary figures**


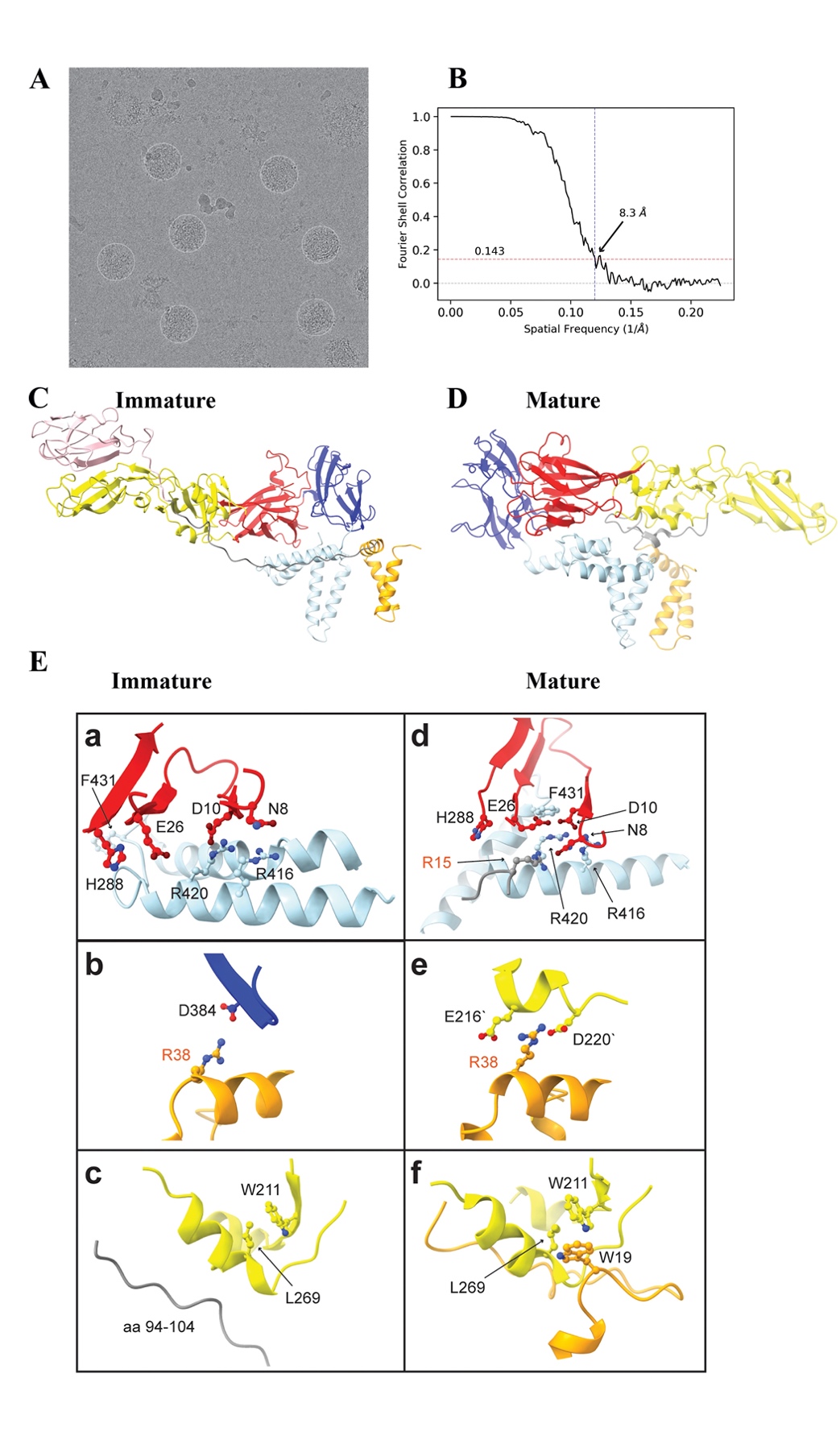


Fig S1

**Fig S1.**

(A) Representative cryo-EM micrograph of the immature ZIKV particles, indicated with white circles. (B) Fourier shell correlation (FSC) curve of the reconstructed map of immature ZIKV particles (shown in Fig. 1), using Gold-standard refinement in cryoSPARC. The approximate map resolution (8.3 Å), based on a 0.143 FSC cutoff, is indicated by an arrow. (C, D) Conformational changes during maturation of ZIKV. Models showing the structure of immature ZIKV (EMD-41037, this study) (C) and mature ZIKV (PDB 6CO8). (D). The domains of the two structures are colored using the convention: pr protein (aa 1-93; pink), the linker region of prM (aa 93-119; grey), M protein (aa 94-168; orange), E D-I (aa 1-51, 136-194, 286-300; red), E D-II (aa 52-135, 195-285; yellow), E D-III (aa 301-402; blue); E C-terminal domain (aa 403-504; light blue). The model of the immature conformation of E protein consists of residues (1-504); the protein domains are indicated as follows: M orange, E D-I red, E D-II yellow, E-DIII blue, E TM helices light blue. The model of prM protein consists of residues (1-168), including the pr domain (pink), M domain (orange), and the linker (grey). (E) Interactions of key amino acids selected for mutagenesis in immature (a, b, and c) and mature conformations (d, e, and f). The M residue numbering is shown in orange.


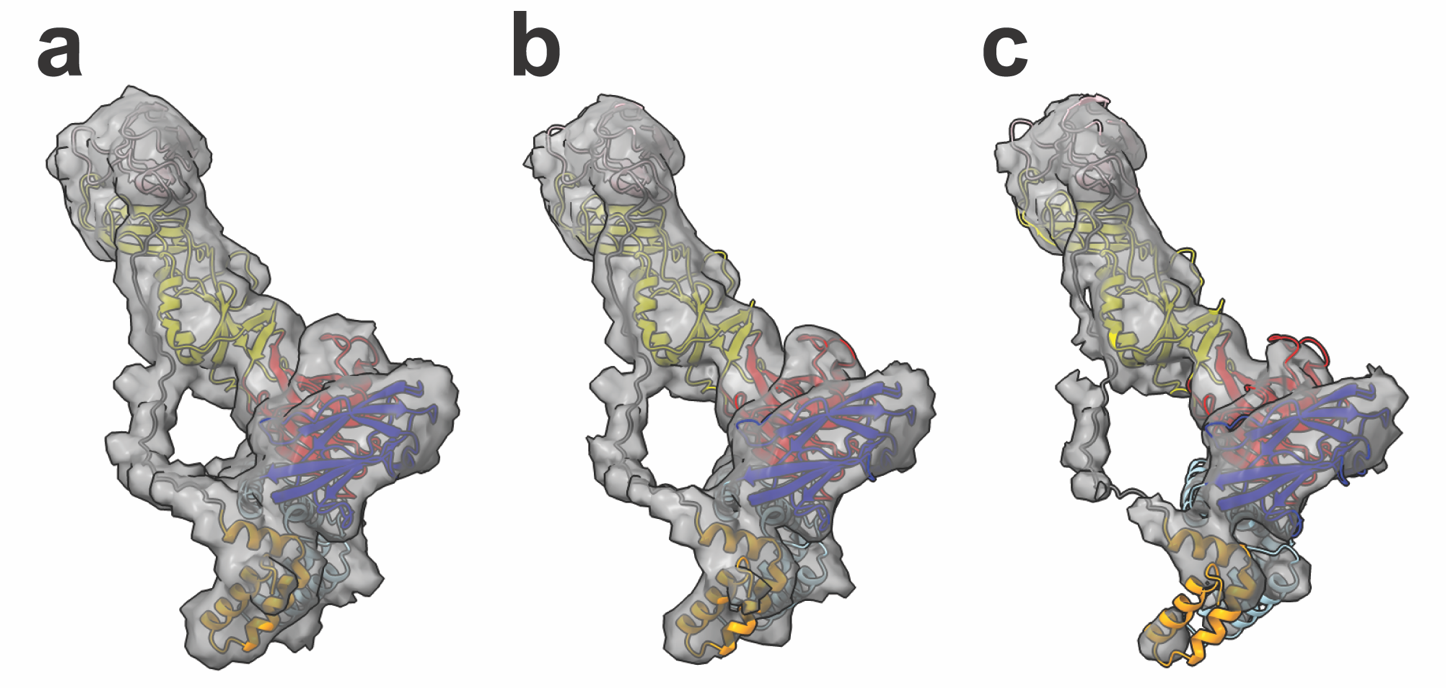


Fig S2

**Fig S2.** **Cryo-EM density map of the prM/E heterodimer comparing the density of the linker region at three contour levels.**

The prM/E heterodimer model fitted into the map, which was contoured at sigma levels **(a)** 2σ , **(b)** 3σ, and **(c)** 4σ.

Fig S3

**Fig S3. Sequence comparison of flavivirus M proteins.**

Multiple sequence alignment of flavivirus M proteins assembled by Clustal Omega and represented by Jalview. The Zappo color scheme was used to differentiate amino acid residues based on physicochemical properties. The arrows indicate amino acids that were substituted with alanine in the study.

Fig S4

**Fig S4. Sequence comparison of flavivirus E proteins.**

Multiple sequence alignment of flavivirus E proteins assembled by Clustal Omega and represented by Jalview. The Zappo color scheme was used to differentiate amino acid residues based on physicochemical properties. The arrows indicate amino acids that were substituted with alanine in the study.


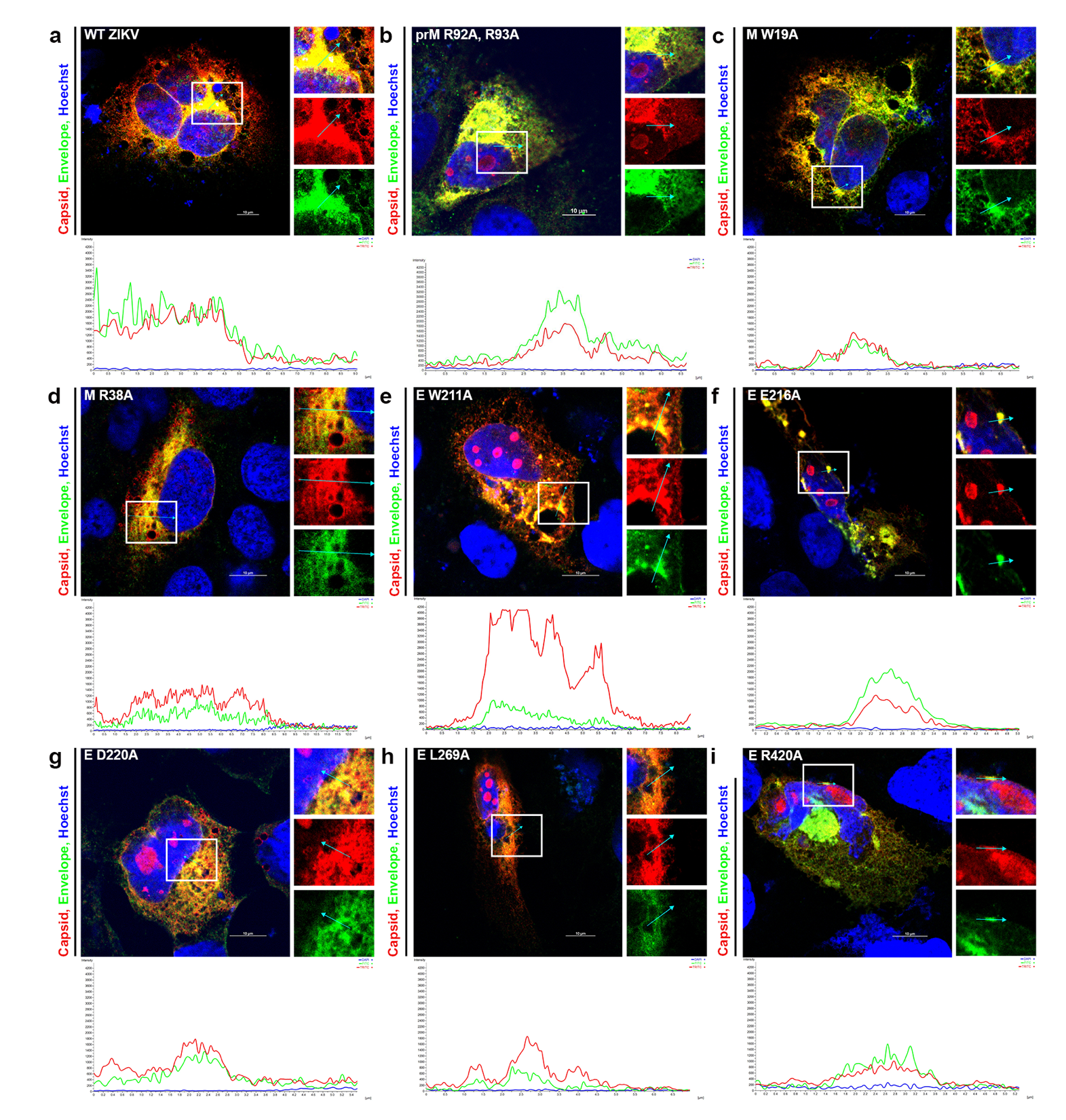


Fig S5

**Fig S5. Immunofluorescence analysis for colocalization of capsid with envelope.**

(a-i) Confocal micrographs of IF assays to detect the colocalization of C protein to E protein. Huh 7.5 cells were transfected with WT and mutant ZIKV cDNAs (a)WT ZIKV, (b) prM R92A, R93A, (c) M W19A, (d) M R38A, (e) E W211A, (f) E E216A, (g) E D220A, (h) E L269A, (i) E R420A, fixed after 36 hours post-transfection, and probed using capsid (red) and envelope (green) antibodies. Regions of interest were analyzed, enlarged to the right, and displayed as merged and separate channels. Analyzed regions are indicated with a cyan arrow, and corresponding profiles are displayed at the bottom, where distance is measured as μm and intensity is measured as arbitrary fluorescence units (AFU).


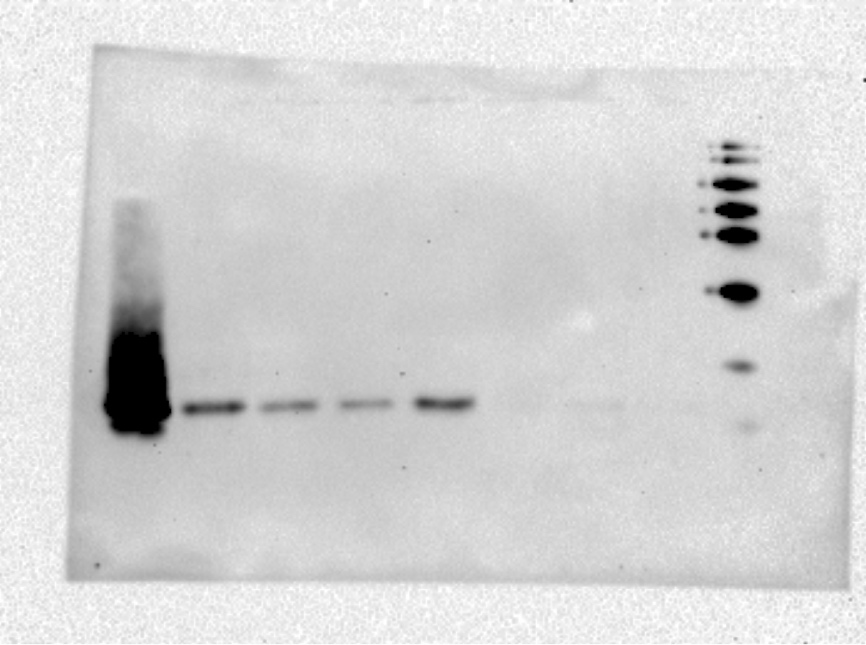


Fig S6

**Fig S6. Full, unmodified western blot image corresponding to Fig. 4b.**

**Supplementary tables**

**Table S1. Primers used for mutagenesis of prM and E proteins.**

| Mutation | Forward (5'-3') | Reverse (5'-3') |
| --- | --- | --- |
| pr E61A | AGGGAGTGGCTCCAGATGATGTCGATTGCTGGTGC | TCATCTGGAGCCACTCCCTCATCCAGCATAGGGCAC |
| pr R92A, R93A | ACGGCGATCTGCCGCCGCCGTGACGCTCCCTTCTCACTCTAC | AGCGTCACGGCGGCGGCAGATCGCCGTGCCTCACCTTTTTTG​ |
| M H7A | TCCCTTCTGCCTCTACAAGGAAGTTGCAAACGCG | TTGTAGAGGCAGAAGGGAGCGTCACGGCTCTTC |
| M R10A | TCTACAGCCAAGTTGCAAACGCGGTCGCAGAC | TTGCAACTTGGCTGTAGAGTGAGAAGGGAGCGTCAC |
| M R15A | AAACGGCCTCGCAGACCTGGTTAGAATCAAG | AGGTCTGCGAGGCCGTTTGCAACTTCCTTGTAGAGTG​ |
| M W19A | AGACCGCCTTAGAATCAAGAGAATACACGAAG | TTGATTCTAAGGCGGTCTGCGACCGCGTTTGCAACTTC |
| M R23A | AATCAGCCGAATACACGAAGCACTTGATCAAG | TCGTGTATTCGGCTGATTCTAACCAGGTCTGCGACC |
| M E24A | ATCAAGAGCTTACACGAAGCACTTGATCAAGGTTG | TTCGTGTAAGCTCTTGATTCTAACCAGGTCTGCGAC​ |
| M Y25A | AGAAGCCACGAAGCACTTGATCAAGGTTGAAAAC​ | AAGTGCTTCGTGGCTTCTCTTGATTCTAACCAGGTCTG​ |
| M H28A | AAGGCCTTGATCAAGGTTGAAAACTGGATATTC​ | AACCTTGATCAAGGCCTTCGTGTATTCTCTTGATTCTAAC |
| M W35A | AAAACGCCATATTCAGGAACCCCGGGTTTGC | TCCTGAATATGGCGTTTTCAACCTTGATCAAGTGCTTC​ |
| M R38A | ATTCGCCAACCCCGGGTTTGCGCTAGTGGC | AACCCGGGGTTGGCGAATATCCAGTTTTCAACCTTGATC​ |
| E E26A | TCTTGGCACATGGAGGCTGCGTTACCGTGATG | AGCCTCCATGTGCCAAGACAACATCAACCCAGGTCC​ |
| E R73A | TCGGACAGTGCCTGCCCAACACAAGGTGAGGCCTAC | TGGGCAGGCACTGTCCGAAGCCATGTCCGATATCG​ |
| E K93A | ATGTCTGCGCAAGAACATTAGTGGACAGAGGTTGG | ATGTTCTTGCGCAGACATATTGAGTGTCTGATTGC​ |
| E Y203A | TCTGTATGCCCTGACCATGAACAATAAGCATTGGTTG | ATGGTCAGGGCATACAGATCTGAAAAGTCAAGGCC |
| E H210A | TAAGGCATGGTTGGTGCACAAAGAGTGGTTTC | TGCACCAACCATGCCTTATTGTTCATGGTCAGGTAATAC​ |
| E W211A | TAAGCATGCCTTGGTGCACAAAGAGTGGTTTCATG | TGCACCAAGGCATGCTTATTGTTCATGGTCAGGTAATAC​ |
| E H214A | TGGTGGCCAAAGAGTGGTTTCATGACATCCC | ACCACTCTTTGGCCACCAACCAATGCTTATTGTTCATG |
| E H216A | AAGCCTGGTTTCATGACATCCCATTGCC | TGTCATGAAACCAGGCTTTGTGCACCAACCAATGCTTATTG |
| E D220A | TGGTTTCATGCCATCCCATTGCCTTGGCATGCTGG | TGGGATGGCATGAAACCACTCTTTGTGCACCAAC​ |
| E E244A | TTGGTAGCCTTCAAGGATGCCCACGCCAAGAG | ATCCTTGAAGGCTACCAATGCCTCTTTGTTGTTCC​ |
| E H249A | TGCCGCCGCCAAGAGGCAAACCGTCGTCGTTC | TGCCTCTTGGCGGCGGCATCCTTGAATTCTACCAATG​ |
| E H266A | AGGAGCCGTTGCCACGGCTCTCGCTGGAGCTCTAG | AGCGAGAGCCGTGGCAACGGCTCCTTCCTGGCTCCCCAG |
| E L269A | TCACACGGCTGCCGCTGGAGCTCTAGAGGCTGAGATG | AGCTCCAGCGGCAGCCGTGTGAACGGCTCCTTCCTG |
| E E274A | TGGAGCTCTAGCCGCTGAGATGGATGGTGCAAAGGG | TCCATCTCAGCGGCTAGAGCTCCAGCGAGAGCCGTGTG​ |
| E R420A | AGGCGCCAAGGCAATGGCAGTCCTGGGGGATACAG | TGCCATTGCCTTGGCGCCTCTCACAGTGGCCTCAAATG​ |
| E W462A | AATGTCCGCCTTCTCACAGATCCTCATAGGCAC | TGTGAGAAGGCGGACATTCCTCCAAACAGTGATTTG​ |
| E W474A | TGCTAGTGGCTTTAGGTTTGAACACAAAGAATGG | AACCTAAAGCCACTAGCAGCGTGCCTATGAGGATC​ |

| Nominal Magnification | x59,000 |
| --- | --- |
| Voltage (keV) | 300 |
| Electron Exposure (e^-^/Å^2^) | 48.3 |
| No. of frames | 54 |
| Defocus range (µm) | -1.2 to -3.0 |
| Pixel size (Å) | 1.11 |
| Symmetry | I1 |
| Initial Particles (no.) | 94,600 |
| Final Particles (no.) | 5,969 |
| Map resolution (Å) | 8.3 |
| FSC Threshold | 0.143 |

**Table S2. Cryo-EM data collection and processing.**

| **Virus** | **Specific infectivity**  **(particles/pfu)** |
| --- | --- |
| Wild type | 10.4 ± 2.8 |
| M R10A | 37.7 ± 4.2 |
| M R15A | 55.2 ± 1.6 |
| M R23A | 57.8 ± 23 |
| E E244A | 487.1 ± 77.3 |

**Table S3. Specific infectivity of plaque-forming mutants.**

Table showing specific infectivity of plaque-forming ZIKV mutants calculated by dividing the number of RNA molecules estimated from the virus particles purified by ultracentrifugation corresponding to 1 ml of the clarified cell-culture supernatants of transfected cells and quantified by qRT-PCR, with plaque forming units (pfu) per ml calculated from the plaque assays. Values represent average specific infectivity with standard error of mean of triplicate samples.
